# Lymphocyte-to-monocyte ratio is associated with prognosis of diffuse large B-cell lymphoma: correlation with CD163 positive M2 type tumor-associated macrophages, not PD-1 positive tumor-infiltrating lymphocytes

**DOI:** 10.18632/oncotarget.14289

**Published:** 2016-12-27

**Authors:** Jingxuan Wang, Kun Gao, Wanting Lei, Lina Dong, Qijia Xuan, Meiyan Feng, Jinlu Wang, Xiangnan Ye, Tuan Jin, Zhongbai Zhang, Qingyuan Zhang

**Affiliations:** ^1^ Department of Medical Oncology, The Third Affiliated Hospital of Harbin Medicial University, Harbin 150040, China; ^2^ Department of Pathology, The Third Affiliated Hospital of Harbin Medical University, Harbin 150040, China; ^3^ Logistics University of People's Armed Police Force, Tianjin 300162, China

**Keywords:** lymphocyte-to-monocyte ratio (LMR), neutrophil-to-lymphocyte ratio (NLR), diffuse large B-cell lymphoma (DLBCL), tumor-associated macrophages, programmed cell death-1 (PD-1)

## Abstract

The research aims to examine the prognostic value of the lymphocyte-to-monocyte ratio (LMR), neutrophil-to- lymphocyte ratio (NLR) and platelet-to-lymphocyte ratio (PLR) in diffuse large B-cell lymphoma (DLBCL). The relation of these hematologic indicators to poor antitumor immunity and prognosis must be investigated. Clinicopathologic data and survival information of 355 patients with DLBCL was retrospectively analyzed. Univariate analysis revealed that lower LMR (<2.71), higher NLR (≥2.81), CD163+ M2 tumor-associated macrophages (TAM) content ≥9.5% and programmed cell death 1 (PD-1)+ tumor-infiltrating lymphocytes (TILs) content < 4.5 cells per high power field(HPF) were significantly related to unfavorable overall survival (OS) and progression free survival (PFS). When considering the prognostic indexes of IPI, multivariate analysis confirmed that LMR of <2.71 and CD163^+^ M2 TAM content ≥9.5% significantly affected the prognosis of DLBCL. Spearman correlation test showed LMR was negatively correlated with CD163^+^ M2 TAM content. However, there were no correlation was found between LMR and PD-1+ TIL as well as between NLR and PD-1+ TIL content. These results indicated that decreased LMR lead to a weak anti-tumor immunity and could be used as a bad prognosis biomarker of DLBCL.

## INTRODUCTION

Diffuse large B-cell lymphoma (DLBCL) is the commonest type of lymphoma, occupying thirty percent to forty percent of preliminary diagnosed non-Hodgkin's lymphomas (NHL), which can be cured with standard immunochemotherapy. Nevertheless, approximately thirty percent patients with late stage of DLBCL remain intractable and the disease could relapse [1]. The International Prognostic Index (IPI),is an evaluating system served as a predictor of the treatment effects in patients with DLBCL; this index is premised on the clinicopathological features of patients [2]. A revised IPI (R-IPI) is added in rituximab plus cyclophosphamide, doxorubicin, vincristine, and prednisone (R-CHOP) therapy showing superior prediction in the outcome of DLBCL patients [3]. Nevertheless, a multitude of patients with different clinicopathological profiles and poor treatment effects remain unestimated.

Studies utilizing gene expression profiling and next-generation sequencing indicate that host inflammatory responses and tumor microenvironment are the defining features of DLBCL [4, 5]. The “stromal-1” signature, which includes genes normally expressed by monocytes and compositions of the extracellular matrix, is associated with satisfactory patient outcome after immunochemotherapy [4]. The cellular components in the tumor immune microenvironment comprise lymphoid cells, mast cells, macrophages, natural killer(NK) cells, dendritic cells and other innate immune cells. Several studies has showed that tumor-associated macrophages (TAM) and peripheral blood monocytes could inhibit host antitumor immunity and affect the prognosis of DLBCL [6–8]. Marchesi et al. found that CD68^+^ TAM content was associated with long term survival, moreover, upregulated the ratio of CD163/CD68^+^ cells and the content of CD163^+^ M2 type TAM, suggestive of M2 polarization of TAMs, which were related to unfavorable prognosis [9]. Nam et al. also suggested that increased M2 TAM content indicates inferior treatment effects for the patients of DBLCL who underwent R-CHOP therapy [8]. Therefore, the effects of CD163^+^ M2 TAM content on the prognosis of DBLCL must be investigated.

Programmed cell death 1 (PD-1), a T cell surface receptor, which belongs to B7 receptor family. Binding of PD-1 to its ligand, namely PD-L1, could block cell-cycle progression in T cells and inhibit cytokine production and is a vital checkpoint in the mediation of immune responses. PD-1 is expressed on tumor-infiltrating lymphocytes (TILs), which are upregulated in various types of solid tumors and related to tumor invasion and unfavorable prognosis.In contrast to solid tumors, the presence of a large number of PD-1^+^ TILs predicts a favorable overall survival (OS) in patients with DLBCL [10–12]. These findings demonstrate that the number of PD-1^+^ TILs reflects not only tumor-mediated T-cell exhaustion but also the origin of lymphoma cells.

Several biological factors, in addition to M2-TAM and PD-1+ TILs, have been recommended as clinical predictors of DLBCL; these prognostic biomarkers are detected by gene expression profiling [13] and immunohistochemistry analysis [14, 15]. However, the predictive significance of these biological markers has not been eventually evaluated; moreover, the methods applied for detection are usually high-priced and hard to implement, and the results are difficult to interpret. Therefore, inexpensive, widely available, and easy to interpreted as prognostic factors in DLBCL must be developed.

Multi-evidence revealed that the ratio of different kinds of peripheral blood cells can be used to predict prognosis of lymphoma. Studies have reported the role of lymphocyte-to-monocyte ratio (LMR), neutrophil-to- lymphocyte ratio (NLR) and platelet-to-lymphocyte ratio (PLR) in predicting the prognosis of various types of malignant lymphoma (ML) [16–18]. Watanabe et al revealed LMR is a simple index that can reflect host systemic immunity and estimate clinical effects of R-CHOP treatments for the patients of DLBCL [16]. Keam et al proved that elevated NLR at diagnosis is an independent indicator of unfavourable prognosis of DLBCL following R-CHOP therapy [17]. Wang et al established a prognostic model at basis of pretreatment PLR and confirmed its usefulness to classify localized extranodal NK-T cell lymphoma into different risk subgroups, which can be used as a guide in selecting treatment modalities [18]. However, no research exist estimating the association of these hematologic prognostic factors and M2 TAM and PD-1^+^ TILs in DLBCL.

This research aims to estimate the prognostic significance of NLR, LMR, and PLR in circulating venous blood. The associations of these factors with the expression of CD163^+^ M2 TAM and PD-1^+^ TILs were also investigated to elucidate DLBCL host immunity and tumor microenvironment. The potential of peripheral blood tests as surrogate biomarker of host immune microenvironment in DLBCL was also determined.

## RESULTS

### Patient characteristics

Clinical characteristics of 355 patients with DLBCL were retrospectively evaluated. It involved 153 females and 202 males, the median age was 54 years (18–86 years). The median OS was 53.71 months (95% confidence interval [CI], 51.11–56.46 months). 124 patients experienced relapse, or disease progression or had died. The 5-year PFS rates and 5-year OS were 65.1% and 74.9%, respectively.

### Cut-off values for ALC, AMC, LMR, and NLR

**Figure 1:**
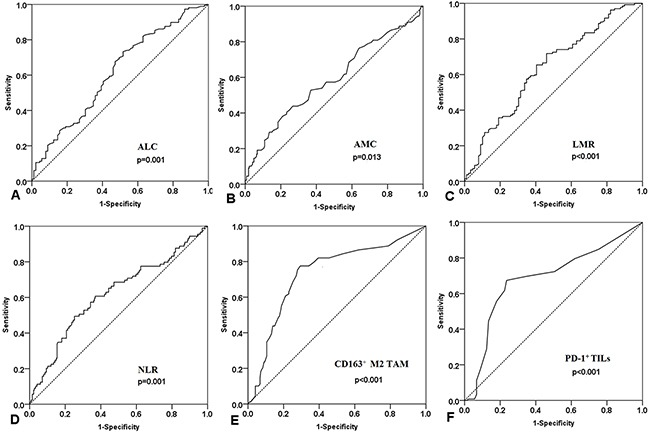
ROC curves analysis for all prognostic factors at diagnosis **A**. ALC (absolute lymphocyte count ). **B**. AMC (absolute monocyte count). **C**. LMR (lymphocyte-to-monocyte ratio). **D**. NLR (Neutrophil-to-lymphocyte ratio). **E**. CD163^+^ M2 TAM (tumor-associated macrophages). **F**. PD-1^+^ TILs (tumor infiltrating lymphocytes).

Althoughwe evaluated the prognostic value of the number of peripheral lymphocytes, monocytes, neutrophils, platelets as well as LMR, NLR and PLR in patients with DLBCL, we didn't make a certain threshold for platelet and neutrophil counts and PLR. Based on the information of survival outcomes, the receiver operating characteristic (ROC) curves were made to determine their cut-off values (Figure 1). The cut-off values for absolute lymphocyte count (ALC) and absolute monocyte count (AMC) were 1.28×10^9^/L and 0.575×10^9^/L.A discriminative cutoff value for LMR was 2.71 (71.8% sensitivity and 53.9% specificity), the area under the curve (AUC) is 0.635 (Figure 1C). Analysis of the ROC curve identified 2.81 (49.4% sensitivity and 74.8% specificity) as the cut-off value for NLR ( AUC = 0.618, Figure 1D).

### Comparison of patient grouping by using the cut-off values for LMR and NLR

Patients were categorized into high-LMR (≥2.71) and low-LMR (<2.71) subgroups or high-NLR (≥2.81) and low-NLR (<2.81) subgroups (Table 1). Differences in OS and PFS were then assessed. A total of 232 patients (65.35%) had LMR ≥ 2.71, and 123 patients (34.65%) had LMR < 2.71. An LMR < 2.71 was significantly correlated with high Ann Arbor stage (p=0.003), increased B symptoms (p<0.001), poor PS (p=0.018), high LDH level (p=<0.001), and presence of numerous extranodal sites (p=0.029). LMR was also relevant to sex (p=0.042), subtype (p=0.004), and CD163 score (p=0.040). A total of 111 patients (31.27%) had NLR ≥ 2.81, and 244 patients (68.73%) had NLR < 2.81. An NLR ≥ 2.81 was significantly correlated with high Ann Arbor stage (p=0.007), B symptoms (p=0.014), poor PS (p=0.040), abnormal LDH level (p<0.001), and bone marrow involved (p=0.003).

**Table 1:** Characteristics of diffuse large B-cell lymphoma according to pre-treatment lymphocyte-to-monocyte ratio and neutrophil-to- lymphocyte Ratio

Characteristic	n (%) n=355	Pre-LMR		P-value	Pre-NLR		P-value
		≥2.71	<2.71		≥2.81	<2.81	
**Sex**				0.042			0.336
**Male**	153(43.1)	109(47.0)	44(35.8)		52(46.8)	101(41.4)	
**Female**	202(56.9)	123(53.0)	79(64.2)		59(53.2)	143(58.6)	
**Age**				0.428			0.912
**≤60 years**	232(65.4)	155(66.8)	77(62.6)		73(65.8)	159(65.2)	
**>60 years**	123(34.6)	77(33.2)	46(37.4)		38(34.2)	85(34.8)	
**Presence of B symptoms**				<0.001			0.014
**No**	308(86.8)	213(91.8)	95(77.2)		89(80.2)	219(89.8)	
**Yes**	47(13.2)	19(8.2)	28(22.8)		22(19.8)	25(10.2)	
**Ann Aarbor stage**				0.003			0.007
**I/II**	222(62.5)	158(68.1)	64(52.0)		58(52.3)	164(67.2)	
**III/IV**	133(37.5)	74(31.9)	59(48.0)		53(47.7)	80(32.8)	
**Performance status**				0.018			0.040
**ECOG 0-1**	343(96.6)	228(98.3)	115(93.5)		104(93.7)	239(98.0)	
**ECOG 2 or more**	12(3.4)	4(1.7)	8(6.5)		7(6.3)	5(2.0)	
**LDH level**				<0.001			<0.001
**Normal**	188(53.0)	150(64.7)	38(30.9)		34(30.6)	154(63.1)	
**Elevated**	167(47.0)	82(35.3)	85(69.1)		77(69.4)	90(36.9)	
**Number of extranodal sites**				0.029			0.231
**0-1**	321(90.4)	215(92.7)	106(86.2)		97(87.4)	224(91.8)	
**2**	23(6.5)	14(6.0)	9(7.3)		8(7.2)	15(6.1)	
**3**	4(1.1)	2(0.9)	2(1.6)		3(2.7)	1(0.4)	
**4-5**	7(2.0)	1(0.4)	6(4.9)		3(2.7)	4(1.6)	
**Bone marrow involvement**				0.846			0.003
**Absence**	319(89.9)	209(90.1)	110(89.4)		92(82.9)	227(93.0)	
**Presence**	36(10.1)	23(9.9)	13(10.6)		19(17.1)	17 (7.0)	
**Bulky disease**				0.415			0.487
**No**	334(94.1)	220(94.8)	114(92.7)		103(92.8)	231(94.7)	
**Yes**	21(5.9)	12(5.2)	9(7.3)		8(7.2)	13(5.3)	
**Subtybe**				0.004			0.231
**GCB**	128(36.1)	96(41.4)	32(26.0)		35(31.5)	93(38.1)	
**Non-GCB**	227(63.9)	136(58.6)	91(74.0)		76(68.5)	151(61.9)	
**CD163+ M2 TAM**	147(41.4)	87(37.5)	60(48.8)	0.040	52(46.4)	95(39.1)	0.194
**PD-1+ TILs**	118(32.2)	78(33.6)	40(32.5)	0.834	35(31.5)	83(34.0)	0.645
**Lymphocyte count (10^9^/L)**	1.74(0.11-4.8)	2.04(0.56-4.8)	1.16(0.11-4.69)	<0.001	1.12(0.11-2.41)	2.02(0.32-4.8)	<0.001
**Monocyte count(10^9^/L)**	0.50(0.08-2)	0.41(0.08-1)	0.68(0.17-2)	<0.001	---------------	---------------	
**Neutrophil count(10^9^/L)**	3.74(0.13-12.5)	---------------	---------------		5.26(1.86-12.5)	3.04(0.13-6.23)	<0.001

Data are shown as n (%) or mean. Abbreviations: GCB, germinal center B cell; pre-LMR, lymphocyte-to-monocyte ratio at diagnose; pre-NLR, Neutrophil-to- lymphocyte ratio at diagnose; TAM, tumor-associated macrophages; TILs, tumor infiltrating leukocytes.

### Cut-off values for CD163+ M2 TAM and PD-1+ TILs

**Figure 2:**
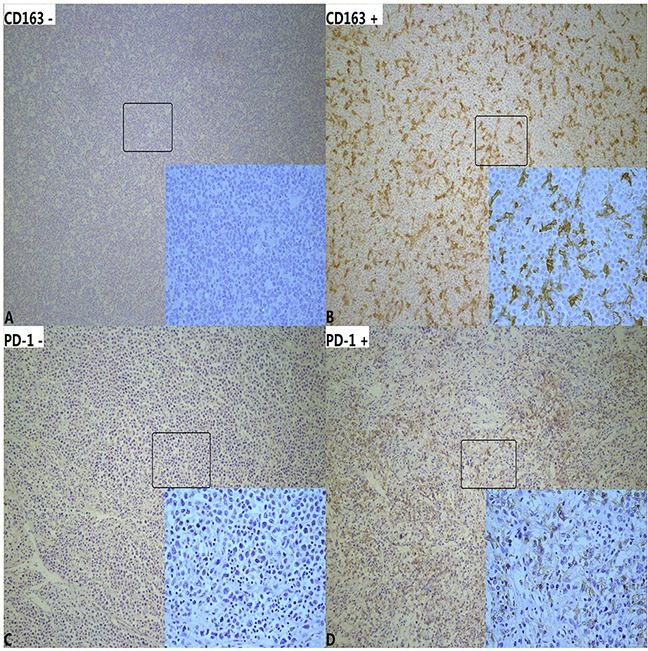
Immunohistochemical staining of tumor-associated macrophages (TAM) and PD-1+ tumor-infiltrating lymphocytes(TILs) in diffuse large B-cell lymphoma (100×HPF and 400×HPF) **A**. Low infiltration of CD163+ M2 TAM (<9.5%). **B**. High infiltration of CD163+ M2 TAM (≥9.5%). **C**. Low infiltration of PD-1+ TILs (<4.5cells/HPF). **D**. High infiltration of PD-1+ TILs (≥4.5cells/HPF).

We evaluated two biologic markers in the tumor microenvironment through immunohistochemical staining and analysis of tumor-associated macrophages expressing CD163 and tumor-infiltrating lymphocytes expressing PD-1 (Figure 2). ROC analysis suggested that CD163^+^ M2 TAM and PD-1^+^ TILs played a role in predicting OS and PFS. For OS, the AUC for CD163^+^ M2 TAM was 0.734 (95% CI 0.671–0.796), indicating 9.5% as the most relevant cutoff value, with a prognostic sensitivity of 77.5% and a specificity of 70.7% (Figure 1E). PD-1+ TIL count could also be used to predict OS and PFS. An optimal cut-off level of 4.5 cells per high-power field (HPF) was selected to evaluate the prognosis between the low and high PD-1^+^ TILs groups, with a prognostic sensitivity of 67.4% and a specificity of 76.4% (Figure 1F).

### Prognostic significance of ALC, AMC, LMR, NLR, CD163+ M2 TAM, and PD-1+ TILs

**Figure 3:**
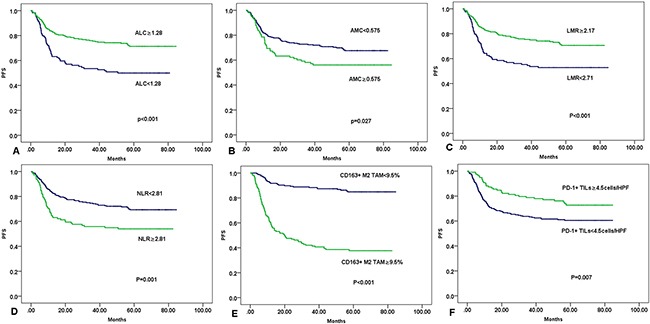
Kaplan-Meier curves of OS for patients by clinical and pathological characteristics **A**. ALC (absolute lymphocyte count). **B**. AMC (absolute monocyte count). **C**. LMR (lymphocyte-to-monocyte ratio). **D**. NLR (Neutrophil-to-lymphocyte ratio). **E**. CD163^+^ M2 TAM (tumor-associated macrophages). **F**. PD-1^+^ TILs (tumor infiltrating lymphocytes).

**Figure 4:**
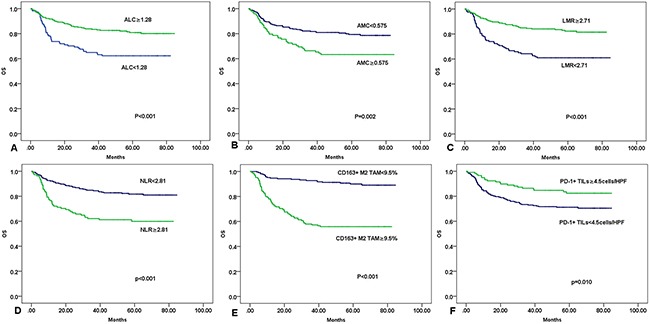
Kaplan-Meier curves of PFS for patients by clinical and pathological characteristics **A**. ALC (absolute lymphocyte count). **B**. AMC (absolute monocyte count). **C**. LMR (lymphocyte-to-monocyte ratio). **D**. NLR (Neutrophil-to-lymphocyte ratio). **E**. CD163^+^ M2 TAM (tumor-associated macrophages). **F**. PD-1^+^ TILs (tumor infiltrating lymphocytes).

Compared to patients with ALC ≥1.28×10^9^/L, patients with ALC < 1.28× 10^9^ /L had significantly lower 5-year PFS rate and 5-year OS rate (5-year PFS rate, 50.5% versus 72.2%; 5-year OS rate, 62.3% versus 80.9%; Figure 3A and 4A, respectively). An AMC value of 0.575×10^9^/L was also significantly associated with low 5-year PFS rate (68.5% versus 56.1%, Figure 3B) and the 5-year OS rate (79.4% versus 63.3%, Figure 4B). Patients with LMR<2.71 had obviously lower 5-year PFS rate than those with LMR ≥ 2.71 (52.85% versus 71.55%, Figure 3C), but their 5-year OS rates were comparable (60.98% versus 82.33%, Figure 4C). NLR is another index of peripheral blood circulation. Higher NLR (≥ 2.81) linked to a worse prognosis (5-year PFS rate, 54.05% versus 78.08%, Figure 3D;and 5-year OS rate, 60.36% versus 81.56%, Figure 4D).

The cut-off scores were used in Kaplan–Meier analysis; the results showed worse OS and PFS for DLBCL cases with ≥ 9.5% CD163^+^ M2 TAM compared with cases with < 9.5% CD163^+^ M2 TAM (5-year PFS rate, 37.9% versus 85.4%, Figure 3E) and OS (5-year OS rate, 55.90% versus 89.80%, Figure 4E). Kaplan–Meier analysis also indicated worse PFS and OS for DLBCL cases with < 4.5 PD-1^+^ TILs cells/HPF compared with subgroups with ≥ 4.5 PD-1^+^ TILs cells/HPF. 5-year PFS rate was 61.5% versus 74.4% (Figure 3F), and 5-year OS rate was 71.8% versus 83.8% (Figure 4F).

The factors influencing OS and PFS are performed through univariate and multivariate analysis (Table 2). The results showed that an LMR < 2.71 was a negative prognostic marker for predicting OS (HR,1.658;95% CI,1.930–2.703; p=0.042) and PFS (HR,1.528; 95%CI, 1.006–2.315; p=0.049). PS ≥ 2 and ≥ 9.5% CD163^+^ M2 TAM were also considered as adverse prognostic factors. Meanwhile, NLR of ≥ 2.81,elevated LDH level, and age > 60 years were associated with poor OS, but not with poor PFS; moreover PD-1^+^ TILs < 4.5 cells/HPF wasn't correlated with OS (p =0.640) or PFS (p=0.410).

**Table 2:** Univariate and multivariate analysis for OS and PFS outcomes

	OS			PFS		
	HR	95%CI	p-Value	HR	95%CI	p-Value
**Univariate analysis**						
**Age >60 years**	1.789	1.171-2.733	0.007	1.332	0.924-1.919	0.124
**Stage III,IV**	1.929	1.264-2.945	0.002	1.790	1.251-2.562	0.001
**ECOG PS≥2**	8.773	4.635-16.61	<0.001	11.64	6.219-21.78	<0.001
**LDH level Elevated**	3.060	1.931-4.850	<0.001	2.114	1.463-3.056	<0.001
**No. of extranodal sites ≥2**	1.658	1.268-2.167	<0.001	1.770	1.392-2.251	<0.001
**Pre-LMR <2.71**	2.907	1.898-4.464	<0.001	2.004	1.401-2.874	<0.001
**Pre-NLR ≥2.81**	2.848	1.864-4.350	<0.001	1.842	1.281-2.649	0.001
**CD163^+^ M2 TAM ≥9.5%**	5.984	3.625-9.879	<0.001	6.288	4.151-9.525	<0.001
**PD-1^+^ TILs <4.5cells/HPF**	1.938	1.164-3.226	0.011	1.727	1.142-2.611	0.010
**Subtybe non-GCB**	1.727	1.063-2.801	0.027	1.783	1.185-2.681	0.006
**Multivariate analysis**						
**Age >60 years**	2.037	1.291-3.214	0.002	1.372	0.937-2.008	0.104
**Stage III,IV**	1.267	0.778-2.066	0.342	1.324	0.877-2.000	0.182
**ECOG PS≥2**	2.829	1.026-7.805	0.045	3.247	1.400-7.527	0.006
**LDH level Elevated**	2.012	1.198-3.377	0.008	1.503	0.984-2.295	0.059
**No. of extranodal sites ≥2**	1.178	0.789-1.757	0.424	1.041	0.757-1.432	0.804
**Pre-LMR <2.71**	1.658	1.930-2.703	0.042	1.528	1.006-2.315	0.049
**Pre-NLR ≥2.81**	1.686	1.036-2.743	0.035	1.174	0.778-1.772	0.444
**CD163^+^ M2 TAM ≥9.5%**	5.387	3.176-9.139	<0.001	5.555	3.610-8.548	<0.001
**PD-1^+^ TILs <4.5cells/HPF**	1.135	0.667-1.934	0.640	1.195	0.782-1.828	0.410
**Subtybe non-GCB**	1.233	0.754-2.016	0.404	1.332	0.874-2.028	0.182

Abbreviations: pre-LMR, absolute lymphocyte/monocyte count ratio at diagnose; pre-NLR, absolute Neutrophil-to-lymphocyte ratio at diagnose; TAM, tumor-associated macrophages; TILs, tumor infiltrating leukocytes; CI, confidence interval; OS, overall survival; PFS, progression-free survival; HR, hazard ratio.

### Correlation between peripheral monocyte count and CD163+ M2 TAM content in tissues

**Figure 5:**
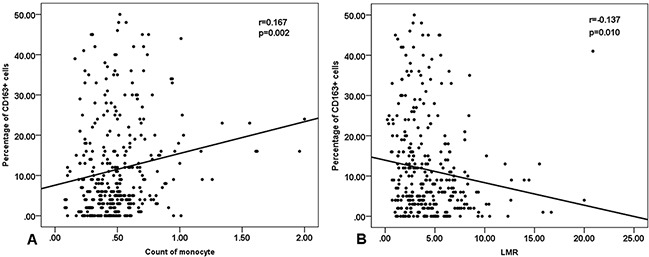
Spearman correlation between peripheral blood monocyte count, lymphocyte-to-monocyte ratio and the number of CD163+ M2 TAM (tumor-associated macrophages) **A**. monocyte count. **B**. LMR (lymphocyte-to-monocyte ratio).

The research confirmed that patients who had a high expression of CD163 (≥9.5%) predicted an unfavorable prognosis. Hence, we conducted a exploring study in order to find their association between the monocyte count and the density of M2 TAM in DLBCL. Spearman correlation analysis indicated a significantly positive correlation between monocyte count in blood and CD163 scores in lymphoma tissues (p=0.002, Figure 5A). The correlation coefficient was 0.167. A negative correlation was found between LMR and CD163 percents in DLBCL tissues (p=0.010, Figure 5B), with a correlation coefficient of −0.137. No correlation was found between PD-1+ TILs and ALC (Figure S1).

## DISCUSSION

Molecular pathology and clinical features are two important factors considered in selecting treatment strategies and predicting DLBCL prognosis. Additional biomarkers must be developed. Studies have established the relationship between the immune system and ML. The pre- and post-treatment amounts of monocytes, neutrophils, and lymphocytes in peripheral blood, which were related to immune system and the prognosis of lymphomas. Studies also reported the prognostic role of LMR, NLR, and PLR in lymphomas and solid tumors [17, 19, 20]. Considering the heterogeneity designs, patient populations and the diversity in treatments had received, we found different LMR and NLR thresholds. In our research, the best thresholds of LMR and NLR are 2.71 and 2.81, respectively, which could be selected to predict the prognosis of DLBCL. Possibly because of the large variations in platelet numbers, we didn't find the threshold for PLR.

The role of LMR in DLBCL varied as reported in several studies. Li et al. confirmed the role of LMR in those patients with DLBCL who received standard first-line treatment regimens [21]. Rambaldi et al. reported the prognostic role of LMR in patients receiving rituximab-based chemotherapy programs [22]. By contrast, Wei et al. confirmed that LMR could predict the prognosis in patients with DLBCL regardless if they undergo rituximab treatments or not [23]. Procházka et al. found that LMR isn't a reliable predictor of the outcome in elderly patients receiving R-CHOP [24]. Our study indicated that low pre-LMR(<2.71) was associated with a high correlation of advanced stage, B symptoms, poor PS, multiple extranodal sites, and high LDH expression. Hence, pretreatment baseline LMR could be added as an independent prognostic factor in DLBCL.

Several studies on the significance of NLR in DLBCL reported similar results. Porrata et al. and Bhumsuk et al. indicated that NLR was an economic, easily, and modeled maker for assessing the prognosis in patients with DLBCL received R-CHOP therapy [17, 19]. Ho et al. believed that ALC/AMC PS could provide incremental prognostic information than LMR and NLR [25]. While, the present study found patients with NLR ≥ 2.81 exhibited high prevalence of high LDH expression, advanced stage, B symptoms, and poor PS. Meanwhile, high NLR was an independent predictor for OS, but not for PFS in multivariate analysis.

The precise mechanism through which low LMR or high NLR results in unfavorable prognosis is unknown. LMR was reported to be negatively associated with the extent of TAM in tumor microenvironment [26], and high pre-NLR was related to low amounts of peripheral NK cells and CD19^+^ lymphocytes [17]. Pathogenesis and survival could be influenced by deficiency of host immunity. A great quantity of TAM, TILs, lymphatic vascular endothelial cells, and other immune cells were detected in tumor stroma. The prognosis of patients with lymphoma were affected by TAM and TILs in tumor stroma [8]. Our study also confirmed the predictive value of >9.5% CD163 ^+^ TAM or > 4.5/HPF PD-1^+^ TILs in determining the prognosis in patients with DLBCL.

An increased number of TAM originating from monocytes, it could advance tumor invasion, transference, and angiogenesis and inhibit antitumor immunity [27]. The quantity of peripheral blood monocytes reflects the formation and/or presence of TAM in lung cancer and colon cancer [28, 29]. Therefore, increased monocyte counts reflect a poor prognosis in patients with cancer or lymphoma. Other studies of DLBCL have shown that high CD68^+^/CD163^+^ M2 TAM or CD163^+^ M2-type macrophage counts at diagnosis were significantly correlated with unfavorable clinical prognosis [8, 9]. Multivariate analysis illustrated that a large index of CD163^+^ M2 macrophages was a reliable prognostic marker of PFS and OS (all p<0.001); moreover, low LMR or high monocytes count was correlated with the high density of CD163^+^ M2-type macrophages. These parameters reflect the associations of host immunity and tumor immune stroma.

Lymphocytes also have an important effects in the passway of antitumor immunity. Because of insufficient antitumor immune, downregulation the amounts of lymphocytes could promote tumor relapse and metastasis [28]. PD-1 maintains immune self-tolerance to avoid autoimmunity and dominates T lymphocyte reaction during infection to avoid excessive tissue damage. A great number of studies had showed that tumor cells escape host antitumor immune assault by the expression of PD-L1 and combination with PD-1 of lymphocytes.PD-1+ immune cells in tumor tissues were significantly associated with unfavorable prognostic factors of solid tumors [30, 31]. In contrast to solid tumors, a high content of PD-1^+^ TILs was related to a well prognosis for patients with DLBCL in our study. Although, no associations were found among LMR, lymphocyte count, and PD-1+ TILs. In tumor microenvironment of solid tumors, activated T and B lymphocytes, progenitor T cells, and NK cells express PD-1. However, in lymphoma, except for activated T cells, follicular helper T (Tfh) cells and the lymphoma cells originated from Tfh cells also express PD-1 [32]. The characteristic molecule of Tfh cells include PD-1, ICOS, as well as the chemokine CXCL13 [32], it promote B cells to form germinal centers. In this study, high levels of PD-1 was found in germinal center B-cell like (GCB) subgroup (Table S2). The number of PD-1+ TILs reflected not only tumor-mediated T-cell exhaustion but also the origin of lymphoma cells. Muenst et al. proved that the decreased amount of PD1+ TILs indicated the transformation of follicular lymphoma into DLBCL [33]. Hartmann et al. found that two patients of DLBCL with high PD-1+ T cell level had an antecedent history of nodular lymphocyte-predominant Hodgkin lymphoma [34]. Ohgami et al. revealed a kind of large B-cell lymphomas was high in T cells but low in B cells; these cells have the similar immunophenotypic characteristics and atypical morphologic as T-cell lymphoma, which with more active PD-1+ T cells [35].

High levels of peripheral neutrophils associates with a worse prognosis of cancer, maybe because of their poor effects on the host. In this study, there were no correlations between the infiltration of CD163^+^ M2 macrophages and PD-1^+^ TILs in DLBCL tissues and the NLR in peripheral blood. Although other studies have shown that high NLR was related to the increasing of monocyte chemotactic protein-1, interleukin-1 receptor α (IL-1R-α), IL-6, IL-7, IL-8, IL-12 and IL-17 in peripheral blood [36, 37], these cytokines could build and keep an immune microenvironment promoting tumor invasion [38]. Hence, high NLR leading to poor DLBCL prognosis may be associated with immune microenvironment. Therefore, future studies should investigate other immune cells in the stroma, for instance, CD4+ T cells, CD8+ T cell and B cell.

The paper is the first research that associate circulating LMR or NLR with PD-1^+^ TILs in the tumor microenvironment of DLBCL; however, the result shows no correlation between LMR and PD-1+ TILs or between NLR and PD-1^+^ TILs. Meanwhile, we have found an inverse correlation between LMR and PD-1^+^ TILs in breast cancer. As surrogate markers of inflammation, LMR or NLR is related to the immune factors, such as TAM in tumor microenvironment, CD19^+^ lymphocytes and NK cells in the peripheral blood [26, 17]. We believe that the nonimmunological factor of PD-1^+^ TILs could explain their different correlation of LMR and PD-1^+^ TILs in solid tumors and DLBCL [39]. Second, peripheral blood monocyte count and LMR are shown to be associated with TAM density in each patient's tumor tissue.

In summary, LMR and NLR are inexpensive clinical parameters that play an essential role in predicting clinical prognosis of DLBCL. LMR, which has more interrelationship with some clinical factors and the infiltration of TAM in tumor microenvironment may be an additional indicator in identifying high-risk patients and predicting whether these patients would benefit from TAM-targeted treatment strategies. However, the factors for assessing efficacy of these therapies are yet to be established. The ideal immune prognostic values of NLR were not found in this study, additional studies are encouraged to validate the correlation of the NLR, LMR, and immune cells in tumor stroma. Because of the limited number of patients, a great number of population is also needed to confirm the best predictive values of these effective and inexpensive tools in the further.

## MATERIALS AND METHODS

### Patients

Retrospectively analyze the data of 355 patients with preliminary confirmed diagnosis DLBCL. These patients received standard treatment in the Tumor Hospital of Harbin Medical University between 2005 and 2011. All patients provided a signed informed consent giving a permission for their medical data. Patients were included if they had: (i) CD20 positive DLBCL which was in accordance with the WHO classification of lymphoid malignancies [1]; (ii) no heart, liver, kidney diseases and other serious somatic diseases; (iii) no other primary malignancy; (iv) available of follow-up records and clinical data.

The data contained patient characteristics, physical examinations, systemic B symptoms, number of extranodal sites contained, bone marrow findings, serum LDH level, Eastern Cooperative Oncology Group (ECOG), biochemical profiles, complete blood count, and thorax, abdomen, and pelvic cavity computed tomography scans or whole-body positron emission tomography/computed tomography (PET-CT) scans. Clinical staging was refered to Ann Arbor staging standards. IPI included Age, Ann Arbor stage, serum LDH, ECOG and extranodal sites[2]. According to Hans’ criteria, the patients were divided into GCB and non-GCB subtypes.

Within 7 days before the first cycle of therapy, pretreatment peripheral blood samples were extracted. The number of different kinds of blood cells was evaluated by XE-2100 hematology analyzer (Sysmex, Kobe, Japan). According to the standard complete blood count results, absolute neutrophil count, ALC, AMC and platelet count were taken. NLR was described by dividing the amount of neutrophils by amount of lymphocytes; LMR was described by dividing the amount of lymphocytes by amount of monocytes;PLR was described by dividing the amount of platelets by amount of lymphocytes.

### Treatment

Patients accepted R-CHOP treatment for 6–8 cycles (day 1: 375 mg/m2 rituximab,50 mg/m2 doxorubicin [adriamycin], 750 mg/m2 cyclophosphamide, 1.4 mg/m2 vincristine [maximum dose 2.0 mg/d] ; 100 mg/ day of prednisone on day1–day 5).

### Follow-up

OS was defined from initial diagnosis until death or the end of follow-up. PFS was estimated from initial diagnosis until progression, death or the end of follow-up. The patient was censored, if the patient was failure to follow-up.

### Immunohistochemistry

Serial sections of 4 μm were utilized for immunohistochemical studies. Immunohistochemical staining for each marker was conducted under the following conditions: CD163 (1:300; Abcam, Cambridge, MA) and PD-1 (1:200; Abcam, Cambridge, MA). These slides were stained overnight, incubated in the secondary antibody solution for 0.5 hour and then viewed through 3,3–diaminobenzidine(DAB)staining. Immunohistochemical sections were evaluated by two pathologists separately. Stained slides were subjected to counterstaining using hematoxylin for improved visualization of the tissue morphology.

CD163+ M2 TAM percentage was determined through the ratio of CD163^+^ M2 TAM to the total number of non-neoplastic cells. The density of PD-1 TILs was evaluated using a hotspot approach, analogous to the previously described method for measuring neoangiogenesis, because of the biopsy size–related dependence of PD-1 TILs [40, 41]. Areas with highest PD-1+ TILs were discovered at low magnification (high power field, 40×) first and then nine areas with the greatest density of PD-1 staining were selected. Next, one 400× magnification was chosen within each hotspot. The final PD-1+ TIL count for an individual was taken as the mean value of the nine counts.

### Statistical analysis

To measure significance between groups of data, unpaired t tests were used, as appropriate. Fisher's exact test or Pearson's χ 2 test was selected for the statistics of categorical variables. Kaplan–Meier was selected to evaluated the effect of different infactors on DLBCL prognosis. Using log-rank test, the survival comparisons of different subgroups were done. The statistical significance was determined by the two-sided p<0.05. A multivariate analysis was analysed by Cox proportional hazards model. ROC curves and AUC were applied to decide the best cutoff points of LMR, NLR, CD163^+^ M2 TAM, and PD-1^+^ TILs. Spearman's rank correlation was applied to estimate the association between quantitative variables. Statistical Package for Social Sciences (SPSS) version 19.0 (SPSS Inc., Chicago, IL, USA) was needed to perform all statistical analyses.

## SUPPLEMENTARY FIGURES AND TABLES


