# Supplementary material for: Lymphocyte-to-monocyte ratio is associated with prognosis of diffuse large B-cell lymphoma: correlation with CD163 positive M2 type tumor-associated macrophages, not PD-1 positive tumor-infiltrating lymphocytes
Source: Oncotarget. 2016 Dec 27;8(3):5414–25. doi: 10.18632/oncotarget.14289 (PMC5354919; doi:10.18632/oncotarget.14289)
Supplement: Supplementary file 1 [file oncotarget-08-5414-s001.pdf]

# Lymphocyte-to-monocyte ratio is associated with prognosis of diffuse large B-cell lymphoma: correlation with CD163 positive M2 type tumor-associated macrophages, not PD-1 positive tumor-infiltrating lymphocytes

## SUPPLEMENTARY FIGURE AND TABLES

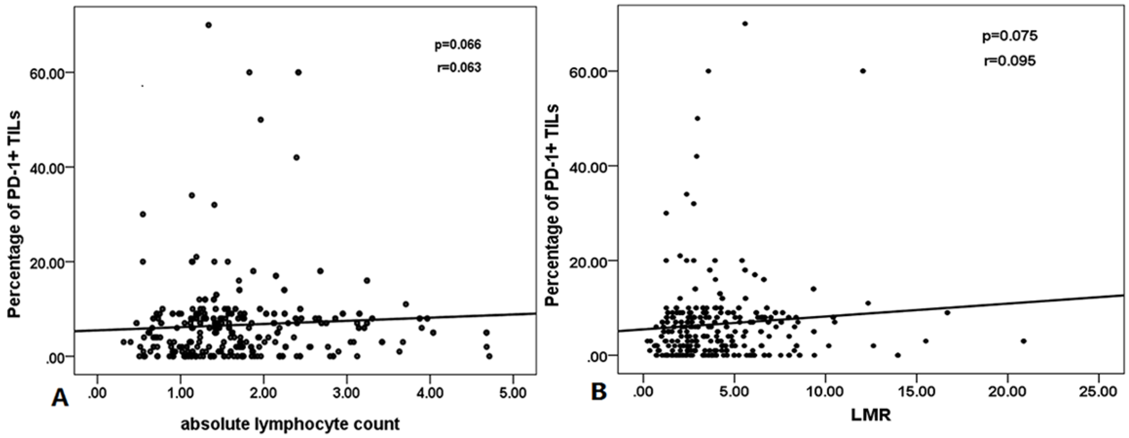


**Supplementary Figure S1: Spearman correlation between peripheral blood lymphocyte count, lymphocyte-to-monocyte ratio and the number of PD-1+ tumor-infiltrating lymphocytes(TILs). A.** lymphocyte count. **B.** LMR (lymphocyte-to-monocyte ratio).

**Supplementary Table S1: Concordance rate of interpretation of immunohistochemistry between visual assessment by two pathologist**

| | Interobserver Agreement (%) | k-value | P |
|---|---|---|---|
| CD163+ M2 TAM | 93.0 | 0.872 | < 0.001 |
| PD-1$^{+}$ TILs | 92.4 | 0.831 | < 0.001 |

TAM, tumor-associated macrophages;PD-1: programmed cell death 1; TIL: tumour infiltrating lymphocytes.

**Supplementary Table S2: Baseline Characteristics of diffuse large B-cell lymphoma patients and correlation with PD-1+ tumor-infiltrating lymphocytes(TILs) on diagnosis**

| Characteristic | n (%) n=355 | PD-1+ TIL | | *P*-value |
|---|---|---|---|---|
| | | ≥4.5cells/HPF | <4.5cells/HPF | |
| **Sex** | | | | 0.760 |
| **Male** | 153(43.1) | 50(41.9) | 103(43.6) | |
| **Female** | 202(56.9) | 69(58.1) | 133(56.4) | |
| **Age** | | | | 0.598 |
| **≤60 years** | 232(65.4) | 80(67.2) | 152(64.4) | |
| **>60 years** | 123(34.6) | 39(32.8) | 84(35.6) | |
| **Presence of B symptoms** | | | | 0.935 |
| **No** | 308(86.8) | 103(86.6) | 205(86.9) | |
| **Yes** | 47(13.2) | 16(13.4) | 31(13.1) | |
| **Ann Aarbor stage** | | | | 0.923 |
| **I/II** | 222(62.5) | 74(62.2) | 148(62.7) | |
| **III/IV** | 133(37.5) | 45(37.8) | 88(37.3) | |
| **Performance status** | | | | 0.525 |
| **ECOG 0-1** | 343(96.6) | 116(97.5) | 227(96.2) | |
| **ECOG 2 or more** | 12(3.4) | 3(2.5) | 9(3.8) | |
| **LDH level** | | | | 0.996 |
| **Normal** | 188(53.0) | 63(52.9) | 125(53.0) | |
| **Elevated** | 167(47.0) | 56(47.1) | 111(47.0) | |
| **Number of extranodal sites** | | | | 0.540 |
| **0-1** | 321(90.4) | 106(89.1) | 215(91.1) | |
| **2-5** | 34(9.6) | 13(10.9) | 21(8.9) | |
| **Bone marrow involvement** | | | | 0.472 |
| **Absence** | 319(89.9) | 105(88.2) | 214(90.7) | |
| **` Presence** | 36(10.1) | 14(11.8) | 22(9.3) | |
| **Bulky disease** | | | | 0.331 |
| **No** | 334(94.1) | 114(95.8) | 220(93.2) | |
| **Yes** | 21(5.9) | 5(4.2) | 16(6.8) | |
| **Subtybe** | | | | <0.001 |
| **GCB** | 128(36.1) | 69(58.5) | 59(24.9) | |
| **Non-GCB** | 227(63.9) | 49(41.5) | 178(75.1) | |

Data are shown as n (%) or mean. Abbreviations: GCB, germinal center B cell; pre-LMR, lymphocyte-to-monocyte ratio at diagnose; pre-NLR, Neutrophil-to- lymphocyte ratio at diagnose; TAM, tumor-associated macrophages; TILs, tumor infiltrating leukocytes.
